# Monovalerin and trivalerin increase brain acetic acid, decrease liver succinic acid, and alter gut microbiota in rats fed high-fat diets

**DOI:** 10.1007/s00394-018-1688-z

**Published:** 2018-04-12

**Authors:** Thao Duy Nguyen, Olena Prykhodko, Frida Fåk Hållenius, Margareta Nyman

**Affiliations:** 1 Food for Health Science Centre, Lund University, Lund, Sweden; 2 Present Address: Department of Food Technology, Engineering and Nutrition, Lund University, PO Box 124, 221 00 Lund, Sweden

**Keywords:** Acetic acid, Caecal microbiota, Succinic acid, SCFA, Valeric acid

## Abstract

**Purpose:**

Short-chain fatty acids (SCFA) are known for their anti-inflammatory properties and may also prevent against the development of metabolic diseases. This study investigated possible effects of two valeric acid esters, monovalerin (MV) and trivalerin (TV) in rats fed high-fat diets.

**Methods:**

Four groups of rats were given a low-fat diet (LF) or a high-fat control diet (HFC) with or without supplementation of MV or TV (5 g/kg) for 3 weeks (*n* = 7/group). SCFA (caecum, blood, liver and brain), succinic acid (liver), microbiota (caecum), lipid profile (liver and blood) and the inflammatory biomarker, lipopolysaccharide-binding protein (blood) were analysed at the end of the experiment.

**Results:**

Supplementation of MV and TV to a high-fat diet increased 1.5-fold the amounts of acetic acid in the brain and 1.7-fold serum concentration of valeric acid, whereas liver succinic acid was reduced by 1.5-fold. Although liver triglyceride levels were higher in both MV and TV groups compared with the LF group, liver LDL/HDL ratio was lower in the MV group (*P* < 0.05). The caecal microbiota composition was altered, with threefold higher abundance of Bacteroidetes and higher ratio of Bacteroidetes/Firmicutes in the MV group compared with the HFC and LF groups. Acetic acid in the brain was negatively correlated with TM7, family S24-7 and *rc4-4*, and positively associated to Tenericutes and *Anaeroplasma*.

**Conclusions:**

The present study shows that MV and TV in the specified dose can affect caecal microbiota composition and, therefore, bacterial metabolites in the liver, serum and brain as well as the lipid profile in the liver.

**Electronic supplementary material:**

The online version of this article (10.1007/s00394-018-1688-z) contains supplementary material, which is available to authorized users.

## Introduction

A high-fat diet, known to induce gut dysbiosis, is associated with an increased risk of various diseases such as type 2 diabetes, inflammatory bowel disease and cardiovascular disease [1–3], while a high consumption of dietary fibre may prevent the onset of these diseases [4]. The changes in gut microbiota composition occur rapidly just in 1 day upon switching diet from low fat, rich in plant polysaccharides, to a Western diet high in fat and sugar [5]. The high-fat diet-induced disturbances in gut microbiota composition may impair the gut defence barrier, allowing influx of inflammatory endotoxins into the circulation, such as lipopolysaccharides (LPS) from Gram-negative bacteria [2], stimulating a cascade of inflammatory responses accompanied with adverse health effects. A typical obese gut phenotype, induced by high-fat diet, is characterised by a decrease in Bacteroidetes and a concomitant increase in Firmicutes, and thus a decreased Bacteroidetes/Firmicutes ratio [6, 7].

The protective effects of dietary fibre and involvement of gut microbiota in inflammatory diseases can be related to anti-inflammatory actions of short-chain fatty acids (SCFA) [8–11]. These molecules, with dominating acetate, propionate and butyrate, are produced from colonic fermentation of dietary fibre. SCFA are well known for their anti-inflammatory effects via activation of their receptors or inhibition of histone deacetylase activity [12, 13]. Acetic acid, the most abundant SCFA, when added to a high-cholesterol diet, has been shown to reduce serum cholesterol and triglycerides in rats [14], and to reduce appetite and food intake by regulating hypothalamic gene expression in the brain of rodents [15].

Another SCFA, valeric acid, formed in small amounts during fermentation of dietary fibre, may also be important in cholesterol metabolism and a valeric acid derivative suppressed cholesterogenesis in rat liver [16]. Notably, the structure of valeric acid is very similar to that of the inhibitory neurotransmitter γ-aminobutyric acid (GABA), except for the terminal amino group. Recently, valeric acid from plant extract was shown to ameliorate dementia in rats by acting as a GABA-agonist [17]. The mechanism of action of valeric acid may be similar to its analogue, valproic acid, which has been shown to increase the production of GABA, resulting in a decreased synthesis of succinic acid [18–20]. Succinic acid, an inflammatory signalling molecule, is elevated in animals subjected to metabolic and inflammatory diseases [21, 22] and in high-fat diets the levels of succinic acid are increased at the expense of butyric acid [23, 24]. To overcome rapid uptake and obtain sufficient release in the gastrointestinal tract, SCFA can be delivered in the form of glycerol esters. Monovalerin (MV) and trivalerin (TV), esters of valeric acid, are used as feed additives with considerable suppressing effects on caecal colonisation of *Salmonella enteritidis* in chickens [25, 26].

The aim of the present study was to investigate whether valeric acid esters, MV and TV, affect the SCFA profile at different sites in the body (portal blood, liver, brain and caecum content) and the composition of the caecal microbiota in conventional rats. To explore possible anti-inflammatory effects of the valeric acid esters, a high-fat diet was used to induce a pro-inflammatory state in the rats. Effects on lipid profile and inflammation were explored by measuring concentrations of total cholesterol, LDL, HDL and triglycerides in serum and liver of rats at the end of the experiment, as well as serum lipopolysaccharide-binding protein (LBP). Furthermore, succinic acid and expression of genes in the liver involved in bile acid synthesis (*Nr0b2, Cyp7a1, Cyp8b1*) were analysed.

## Materials and methods

### Study design, diets and animals

The present study was approved by the Local Ethical Review Committee for animal experiments in Lund, Sweden (approval number M295-12).

The study was designed to test four diets: three high-fat diets where two were supplemented with 5 g/kg (dry weight basis) of MV and TV, respectively, (Perstorp AB, Sweden) and a third diet with no valerins, referred to as control (HFC). The fourth diet was a diet containing low fat (LF). All diets were prepared as described in Supplementary Table S1.

Twenty-eight conventional male Wistar rats (Taconic, Denmark) with an initial average weight of 146 g (SEM 2), were randomly divided into four groups of seven. Each group was housed into two cages (three or four rats per cage) in a controlled environment (21 °C, 12 h light–12 h dark cycle). After 3 days of acclimatisation to the environment, the animals had free access to the test diets and water during the following 3-week experimental period. New portions of food were added every 2–3 days, and residues were weighed and recorded accordingly. Rat body weights were recorded weekly at the same time point.

The rats were fed with the testing diets until dissection. On the dissection day, prior to tissue collection, the rats were anaesthetised by a subcutaneous injection mixture (1:1:2) of Hypnorm (Division of Janssen-Cilag Limited, Janssen Pharmaceutica, Beerse, Belgium), Dormicum (F. Hoffmann-La Roche AG, Basel, Switzerland) and autoclaved Milli-Q Millipore water, at a dose of 0.15 ml/100 g body weight. Blood samples, collected from the hepatic portal vein, were placed immediately in serum tubes (SST™ II Advance, Plus Blood Collection Tubes, BD Vacutainer, Plymouth, UK), and directly analysed for blood glucose (HemoCue^®^ Glucose 201^+^ Analyzer from HemoCue AB, Ängelholm, Sweden). After centrifugation, the obtained serum supernatants were stored at − 40 °C until analysis of SCFA, LBP, cholesterol and triglycerides. The caecum was removed and weighed with and without its content. Caecal tissue was washed with Milli-Q Millipore water, while content was subjected to pH measurement before being stored at − 20 °C for the analysis of SCFA and gut microbiota composition. Other organs (liver, spleen, stomach, small intestine and colon) were removed, weighed and stored at − 80 °C for any further analysis. SCFA and succinic acid were analysed in the liver.

### Analyses

#### Carboxylic acids

SCFA in caecal content and freeze-dried liver samples were measured by a methodology using direct injection gas chromatography [27] after being extracted with acidified water. In serum and brain samples, concentrations of SCFA were pre-enriched and extracted by hollow fibre before being injected and analysed with gas chromatography [28].

Succinic acid in the liver was measured by ion-exclusion chromatography as previously described [23].

#### Cholesterol and triglycerides in blood and liver

Lipids in freeze-dried liver samples were extracted using a modified method with low-toxicity solvent [29], as previously described [23].

#### LBP

LBP concentrations in serum were determined using an LBP ELISA kit, following the manufacturer’s instructions (Hycult Biotech, Uden, The Netherlands).

#### Caecal microbiota

Extraction of DNA was performed from 50 to 100 mg of rat’s caecum content using the QIAamp Fast DNA Stool Mini Kit (Qiagen, Hilden, Germany), according to the manufacturer’s protocol using an additional bead-beating step. DNA concentration was measured using Qubit 2.0 Fluorometer (Life Technologies, Sweden).

DNA was amplified using primers for the target gene 16S rRNA (V3-4 regions: forward 5′-CCTACGGGNGGCWGCAG-3′ and reverse 5′-GACTACHVGGGTATCTAATCC-3′). The preparation of the amplicon library was done according to 16S Metagenomic Sequencing Library Preparation protocol (https://www.illumina.com). The sequencing of the library with a read length of 2 × 300 bp using Miseq v.3 reagent kit (the batch produced in 2014) was carried out on a Miseq Instrument (Illumina Inc., San Diego, USA).

Sequencing data (FASTQ format) were analysed using an open-source bioinformatic pipeline, Quantitative Insights into Microbial Ecology (QIIME, v 1.9.1). Forward and reverse reads were joined and then quality filtering was performed. As a result, a total number of 3,817,935 reads were generated for 23 samples (LF, *n* = 6; HFC, *n* = 5; MV, *n* = 6; TV, *n* = 6) with a mean of 165,997 reads per sample (min: 36,478 and max: 312,813). The sequences were grouped into operational taxonomic units (OTUs) at a minimum of 97% similarity, generating 7 OTUs at phylum level and 33 OTUs at genus level. Taxonomy was assigned using the Greengenes database (v.13.8). Alpha rarefaction to estimate alpha diversity was applied to the OTU table at an even depth of 36,400 sequences per sample, thus including all samples in the analysis.

#### Gene expression

PrimePCR for *Nr0b1, Cyp7a1* and *Cyp8b1* was purchased from Bio-Rad (California, USA). Gene-specific primers were added in a pre-made PrimePCR plate from Bio-Rad (Solna, Sweden). Total RNA in freeze-dried liver tissues (approximately 30 mg) was purified using the RNeasy Mini Kit with on-column DNase digestion (Qiagen GmbH, Hilden, Germany), and converted to cDNA using the Thermo Scientific™ RevertAid™ First Strand cDNA Synthesis Kit (Thermo Fisher Scientific). The quantitative real-time PCR was performed using a Bio-Rad T100 Thermal Cycler with SYBR Green SsoAdvanced Universal Supermix (Bio-Rad, California, USA). The amplification protocol was as followed: activation at 95 °C for 2 min, followed by 40 cycles of 5 s denaturation at 95 °C, and annealing at 60 °C for 30 s. Specificity of the PCR products was checked by performing a melt curve analysis. GAPDH was used as a reference gene. The ∆∆Ct method was used to calculate relative mRNA expression.

### Statistical evaluation

GraphPad Prism 7.03 was used to analyse the data. D’Agostino–Pearson test was used to check normality of the data. One-way ANOVA, followed by Dunnett’s method, was applied to specifically identify which groups were significantly different from the LF control group. Furthermore, to evaluate whether the valerins could counteract the adverse effects induced by high-fat feeding, these diets were also compared with the HFC group, to validate the effects of the valerins. Thus, the group fed the LF diet was used as reference (assumed to be the basic value), and the group fed the HFC diet generated the worst values. For nonparametric data, Kruskal–Wallis test was used. For microbiota analysis, the same statistical approach was used to find differences in relative abundances, at phylum and genus level, and to correct for multiple comparisons. Projection-to-Latent-Structures-Discriminant Analysis (PLS-DA) (SIMCA version 14, Umetrics, Umeå, Sweden) was employed to analyse and visually display connection between the gut microbiota data and variables showing significant differences. *P* values < 0.05 were statistically significant, while 0.2 ≥ *P* values ≥ 0.05 were considered as tendency. Results are presented as means and their standard errors (SEM).

## Results

### Body weight and tissue weights

All rats remained active and healthy during the experiment. No difference was seen in final body weight, body weight gain and weights of liver, caecal tissue and content, nor in caecal pH and blood glucose levels between the groups (Table 1). However, rats in the MV group had lower spleen weight than rats in the HFC (*P* = 0.086) and LF (*P* = 0.022) groups.

**Table 1:** Final body weight, body weight gain, total food intake, food efficiency ratio (body weight gain/food intake), actual and relative weights of liver, spleen, and caecal tissue, caecal content and pH and blood glucose in rats fed a low-fat (LF) diet, a high-fat control (HFC) diet or the HFC diet supplemented with MV or TV for 3 weeks

Weights/groups	LF		HFC		MV		TV	
	Mean	SEM	Mean	SEM	Mean	SEM	Mean	SEM
Final body weight (g)	292	8	270	4	268	10	268	5
Body weight gain (g)	137	6	120	3	128	8	129	5
Total food intake (g/rat)	453	2	417^†^	2	330*^†^	3	336*^†^	6
FER (g/g)	0.30	0.01	0.29	0.01	0.39***^††^	0.02	0.39***^††^	0.02
Liver (g)	11	0.4	10	0.4	10	0.4	10	0.3
Relative liver (mg/g bw)	37.5	0.1	38.1	0.2	37.3	0.1	38.0	0.1
Spleen (g)	0.64	0.03	0.61	0.03	0.52^†^	0.02	0.60	0.04
Relative spleen (mg/g bw)	2.18	0.1	2.27	0.1	1.95	0.0	2.23	0.1
Caecal tissue weight (g)	0.47	0.02	0.48	0.02	0.53	0.02	0.52	0.03
Relative caecal tissue (mg/g bw)	1.63	0.074	1.78	0.000	1.97^†^	0.057	1.93^†^	0.094
Caecal content (g)	1.7	0.2	2.2	0.3	1.7	0.2	1.7	0.3
Caecal pH	7.4	0.07	7.5	0.06	7.5	0.10	7.4	0.07
Blood glucose (mmol/l)	8.4	1.0	8.1	1.2	8.0	0.8	8.0	0.8

Values are mean ± SEM, *n* = 7

FER, food efficiency ratio; bw, body weight

Mean values were significantly different from that of the HFC group: *P < 0.05, **P < 0.01, ***P < 0.001

Mean values were significantly different from that of the LF group: ^†^*P* < 0.05, ^††^*P* < 0.01

MV and TV groups had higher relative caecal tissue weights (*P* < 0.05) than the LF group, while the final body weights tended to be lower for these groups (*P* = 0.063 and *P* = 0.073 for MV and TV, respectively). However, when the food intake was considered, the body weight gain (food efficiency ratio, FER) in rats fed MV and TV diets was higher than in rats fed the control groups (HFC *P* < 0.001 and LF diet *P* < 0.01).

### Monovalerin and trivalerin influence SCFA profile at different locations

#### Caecum

The total caecal amount of SCFA was lower in the HFC group (82 µmol) than in LF group (100 µmol). Addition of MV and TV to a HFC diet decreased the caecal amount of SCFA even more. The decrease was significantly compared with the group fed the LF diet but not compared with the HFC group (Table 2, 65 and 67 µmol for MV and TV, respectively, vs. 100 µmol for the LF diet, *P* < 0.05). A similar decrease could be seen with the specific SCFA and it was significant for acetic, propionic and valeric acids (only MV) (*P* < 0.05 to *P* < 0.01).

**Table 2:** SCFA in the caecum, portal serum, liver and brain of rats fed a low-fat (LF) diet, a high-fat control (HFC) diet or the HFC diet supplemented with MV or TV for 3 weeks

	LF		HFC		MV		TV	
	Mean	SEM	Mean	SEM	Mean	SEM	Mean	SEM
*Caecal pool* (µmol)								
Total	100	11	82	10	65^†^	5	67^†^	7
Acetic acid	71	8	57	7	47^†^	4	49^†^	6
Propionic acid	16	2	11^†^	1	8^††^	1	8^††^	1
Butyric acid	9	2	9	1	6	1	7	1
Valeric acid	1.7	0.2	1.5	0.2	1.0^†^	0.2	1.1	0.1
Iso-butyric acid	1.5	0.2	1.2	0.3	0.9	0.2	1.0	0.2
Iso-valeric acid	1.3	0.2	1.3	0.2	1.0	0.1	1.1	0.2
*Portal serum* (µmol/l)								
Total	743	32	753	80	788	62	783	59
Acetic acid	628	35	635	64	660	48	658	47
Propionic acid	47	2	45	6	38	6	34	5
Butyric acid	32	2	38	6	52	8	46	6
Valeric acid	9	2	13	2	22^†^	3	26**^††^	3
Iso-butyric acid	13.8	1	12.5	1	9.6	1	9.1^†^	1
Iso-valeric acid	14.5	3	10.7	2	9.4	2	9.3	2
*Liver* (µmol)								
Total	305	33	149^†^	26	244	29	246	52
Acetic acid	288	31	136^†^	24	224	25	232	49
Propionic acid	5	0.4	4	0.5	5	0.5	5	0.9
Butyric acid	3	0.2	2	0.1	3	0.3	2	0.1
Valeric acid	1.1	0.2	0.5^†^	0.1	0.7	0.1	0.8	0.1
Iso-butyric acid	0.05	0.01	0.08	0.01	0.15	0.04	0.08	0.04
Iso-valeric acid	9	3	5	1	10	3	5	2
Caproic acid	0.6	0.04	0.5	0.07	0.6	0.06	0.4	0.06
Heptanoic acid	0.4	0.04	0.6	0.09	0.5	0.06	0.5	0.09
*Brain* (µmol)								
Total	157	16	135	11	204**	15	220***^††^	11
Acetic acid	153	16	131	11	199**	15	216***^††^	10
Propionic acid	2.6	0.3	2.8	0.5	3.7	0.6	3.6	0.6
Butyric acid	0.06	0.01	0.08	0.02	0.05	0.02	0.07	0.01
Iso-butyric acid	0.3	0.05	0.3	0.07	0.5	0.06	0.5	0.09
Iso-valeric acid	0.45	0.04	0.50	0.07	0.32*	0.02	0.38	0.02

Values are mean ± SEM, *n* = 7

Mean value was significantly different from that of the HFC control group: *P < 0.05, **P < 0.01, ***P < 0.001

Mean value was significantly different from that of the LF group: ^†^*P* < 0.05, ^††^*P* < 0.01

#### Serum

There were no significant differences in the concentration of acetic, propionic- or butyric acids between groups (Table 2). However, the concentration of valeric acid in the TV group was twice as high as the concentration in the HFC group (26 vs. 13 µmol/l, *P* = 0.007). Furthermore, both MV and TV groups had higher concentrations of valeric acid compared with the LF group (*P* < 0.05 and *P* < 0.01, respectively).

#### Liver

Rats fed the LF diet had considerably higher amounts of total (*P* = 0.014) and some specific SCFA (acetic acid *P* = 0.012, and valeric acid *P* = 0.017) than rats fed the HFC diet, which revealed the lowest amounts (Table 2). Supplementation with MV and TV to HFC diet increased the amount of total and specific SCFA in these groups but did not reach the same levels as with the LF group. MV and TV groups revealed very similar levels.

#### Brain

Total amounts of SCFA were higher in MV and TV groups, compared with the groups fed LF (*P* < 0.01 for TV) and HFC (*P* < 0.01 for MV and *P* < 0.001 for TV). Acetic acid was found in highest proportions in the brain samples and the amounts were significantly higher with MV and TV groups compared with the HFC group (*P* < 0.01), and for TV also compared with the LF group (*P* < 0.01). The amount of iso-valeric acid was lower in the MV group than in the HFC group (*P* < 0.05). Valeric acid was not detected in the brain with any groups.

### Monovalerin and trivalerin reduce hepatic succinic acid concentration

**Fig. 1:**
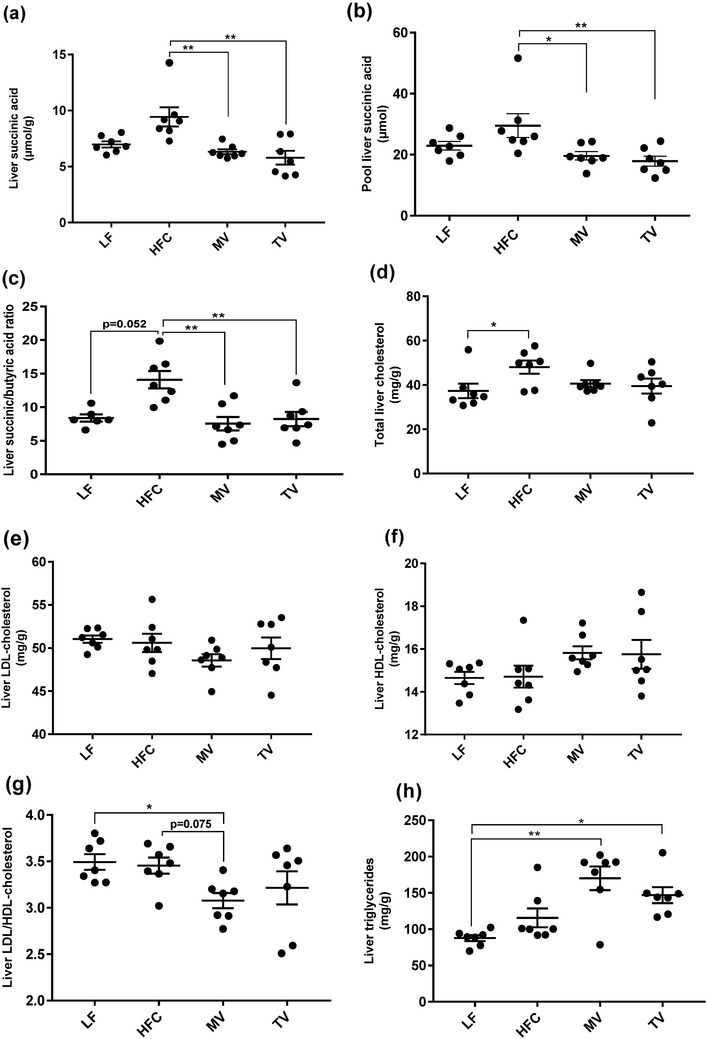
Liver succinic acid and lipid concentrations of rats fed a low-fat (LF) diet, a high-fat control (HFC) diet or the HFC diet supplemented with MV or TV for 3 weeks. **a** Succinic acid (µmol/g), **b** pool succinic acid (µmol), **c** succinic/butyric acid ratio, **d** total cholesterol (mg/g), **e** LDL-cholesterol (mg/g), **f** HDL-cholesterol (mg/g), **g** LDL-to-HDL-cholesterol ratio, **h** triglycerides (mg/g). Values are means ± SEM. Mean values were significantly different from the HFC or LF group: *P < 0.05, **P < 0.01

The concentration of succinic acid in the liver was lower in the MV and TV groups compared with the HFC group (Fig. 1a,* P* = 0.023 and *P* = 0.003, respectively) and it was quite similar as with the group fed the LF diet. Similar results could be seen with the total amount of succinic acid (Fig. 1b, * P*= 0.023 and *P* = 0.003, respectively). Interestingly, the ratio between succinic and butyric acid was also higher in the HFC group (14.1) compared with MV, TV and LF groups (Fig. 1c, 7.5, 8.2 and 8.4, respectively, *P* < 0.05).

### Lipid profile

#### Liver

Total cholesterol concentrations in the HFC group were higher than in the LF group (48 vs. 37 mg/g, *P* = 0.039) (Fig. 1d). Supplementation with MV and TV tended to lower cholesterol concentration to 41 and 39 mg/g, respectively (*P* = 0.130 and* P* = 0.076, respectively). There was no significant difference in LDL and HDL concentrations between groups (Fig. 1e, f), but the ratio of LDL/HDL was lower in the MV group compared with both the HFC and LF group (Fig. 1g, P = 0.075 and *P* = 0.045, respectively). Triglyceride concentrations were higher in MV (*P* < 0.01) and TV (*P* < 0.05) groups compared with the LF group, and similar to the HFC group (Fig. 1h).

#### Blood

**Fig. 2:**
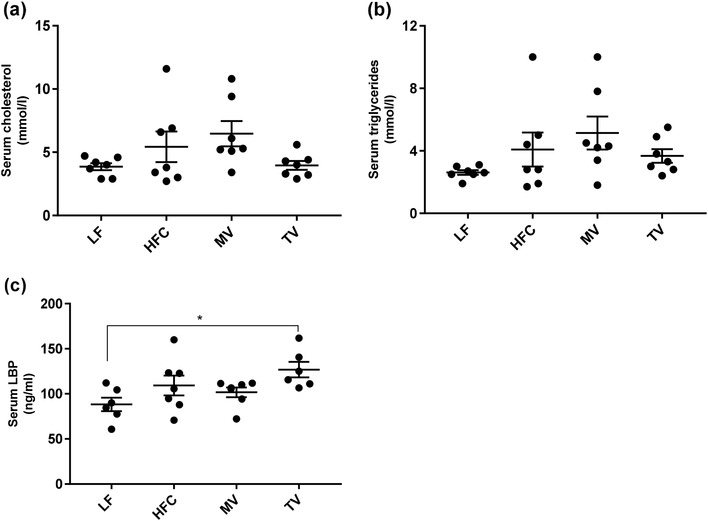
Serum lipid and LBP concentrations of rats fed a low-fat (LF) diet, a high-fat control (HFC) diet or the HFC diet supplemented with MV or TV for 3 weeks. **a** Total cholesterol (mmol/l), **b** triglycerides (mmol/l), **c** LBP (ng/ml). Values are means ± SEM. Mean values were significantly different from the LF group: *P < 0.05

No significant difference was found in serum cholesterol or triglyceride concentrations between the groups (Fig. 2a, b).

### LBP

The serum concentrations of LBP did not differ significantly between the HF groups. However, the TV group had higher LBP concentrations than the LF group (Fig. 2c,* P* = 0.014).

### Hepatic gene expression

The relative expression of *Nr0b2, Cyp7a1* and *Cyp8b1* genes was similar among all groups (Supplementary Table S2).

### Caecal microbiota

#### Phylum level

**Fig. 3:**
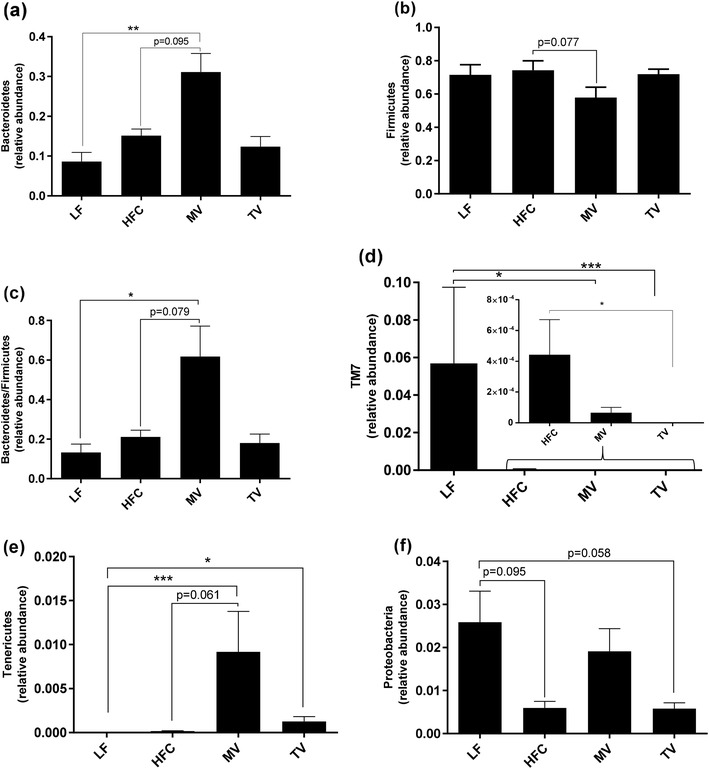
Caecal microbiota composition (phylum level) of rats fed a low-fat (LF) diet, a high-fat control (HFC) diet or the HFC diet supplemented with MV or TV for 3 weeks. Values are means ± SEM, where * indicates significant difference between groups: *P < 0.05, **P < 0.01, ***P < 0.001

The relative abundance of Bacteroidetes was higher in the MV group than in the LF group (*P* = 0.002) and with a tendency also compared with the HFC group (*P* = 0.095) (Fig. 3a), while the abundance of Firmicutes tended to be lower in the MV group (*P* = 0.077 compared with the HFC group) (Fig. 3b). This resulted in a higher ratio of Bacteroidetes-to-Firmicutes with the MV group than with HFC and LF groups (Fig. 3c, * P*= 0.079 and *P* = 0.011, respectively).

Furthermore, the LF group had a considerably higher abundance of TM7 than HFC group (128 times, * P*= 0.043). This amount decreased with MV supplementation (Fig. 3d, *P *= 0.009 compared with the LF group), and in the TV group this phylum could not be detected at all (*P* < 0.001).

Tenericutes phylum was not detected in the LF group and very low in the HFC group, increased when supplementing the diet with MV and TV (Fig. 3e,* P* < 0.001 and *P* = 0.018 compared with the LF group, respectively) and tended to be higher than the HFC group (*P* = 0.061) for MV. Proteobacteria tended to be higher in the LF group compared with the HFC group (Fig. 3f,* P* = 0.095). MV and TV supplementation did not change this distribution to any greater extent.

#### Genus level

**Fig. 4:**
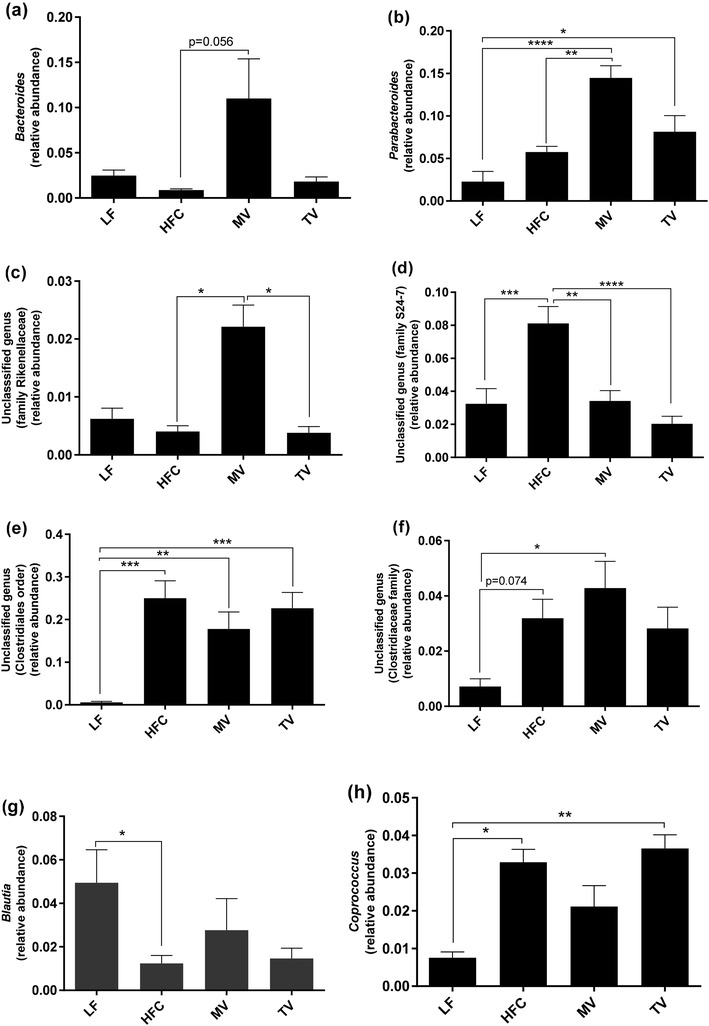

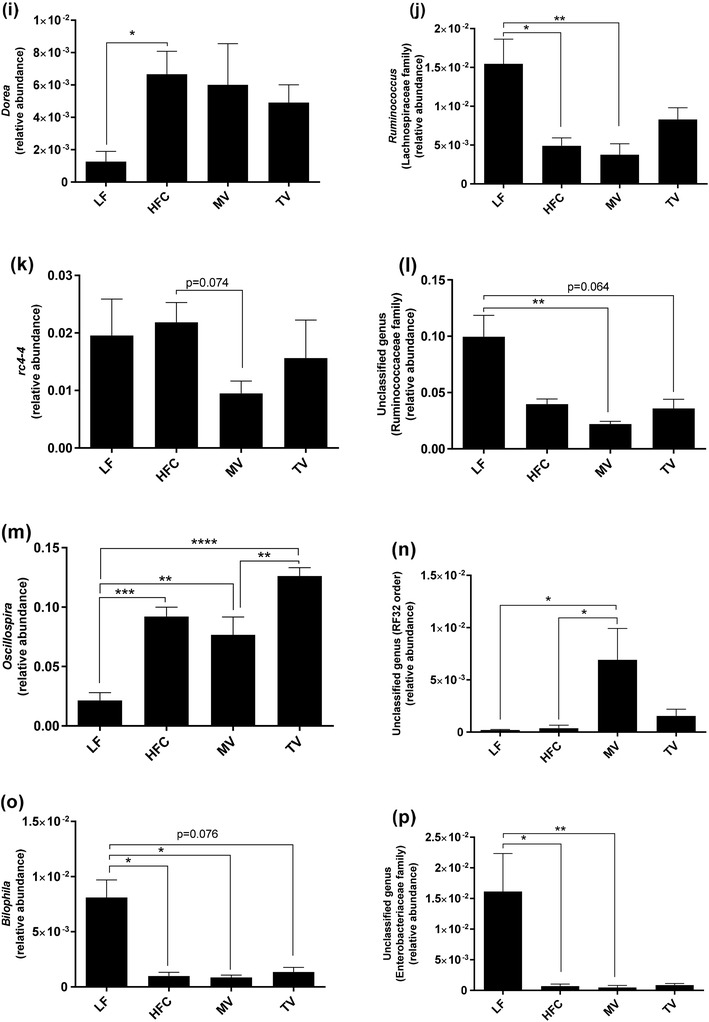

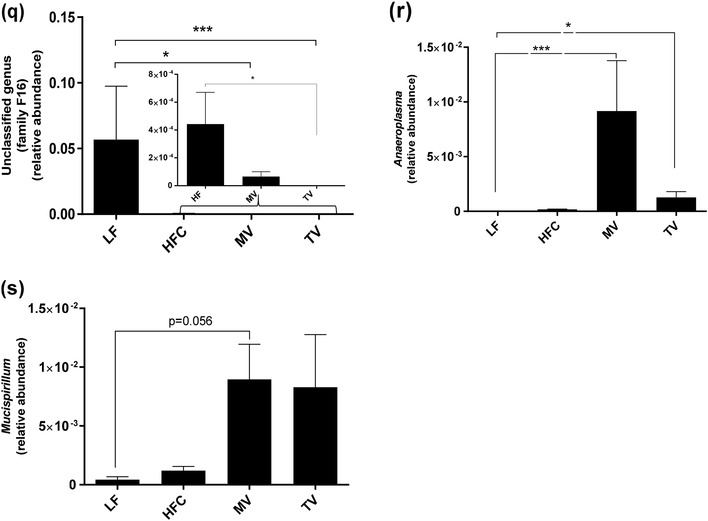
Caecal microbiota composition (genus level) of rats fed a low-fat (LF) diet, a high-fat control (HFC) diet or the HFC diet supplemented with MV or TV for 3 weeks. Values are means ± SEM, where * indicates significant difference between groups: *P < 0.05, **P < 0.01, ***P < 0.001, ****P < 0.0001

Changes at the phylum level were also reflected at genus level (Fig. 4). The relative abundance of *Blautia, Ruminococcus* (Lachnospiraceae family) and *Bilophila* was lower in the group fed the HFC diet than the group fed the LF diet, while the abundance of unclassified genera in the order Clostridiales and family Clostridiaceae, *Coprococcus, Dorea*, and *Oscillospira* (*P* < 0.05) was higher. Supplementation of MV and TV to the HFC diet did not change the abundance of these genera compared to the group fed the HFC diet. However, the relative abundance of some other genera (*Bacteroides, Parabacteroides*, an unclassified genus—family Rikenellaceae) was stimulated by MV and this group had higher abundance of these bacteria than the HFC group (0.01 < *P* < 0.058). Furthermore, the abundance of an unclassified genus (RF32 order) was higher in MV group (*P* < 0.05) compared with the HFC and also the LF group. On the other hand, *rc4-4* tended to be lower (*P* = 0.074) in the group fed the MV diet.

No difference could be seen in any genera concerning the group fed the TV diet compared with the HFC group, except for the lower relative abundance of an unclassified genus (family S24-7, *P* < 0.001). The group fed the MV diet also had significantly lower abundance of this genus.

Compared with the LF diet, the relative abundance of an unclassified genus (family F16) was lower with MV and TV (*P* < 0.05 and *P* < 0.001, respectively), while the abundance of *Anaeroplasma* was higher in MV and TV groups (*P* < 0.001 and *P* < 0.05, respectively). Furthermore, *Mucispirillum* abundance tended to be higher in the MV group (*P* = 0.056).

### Multivariate analysis

**Fig. 5:**
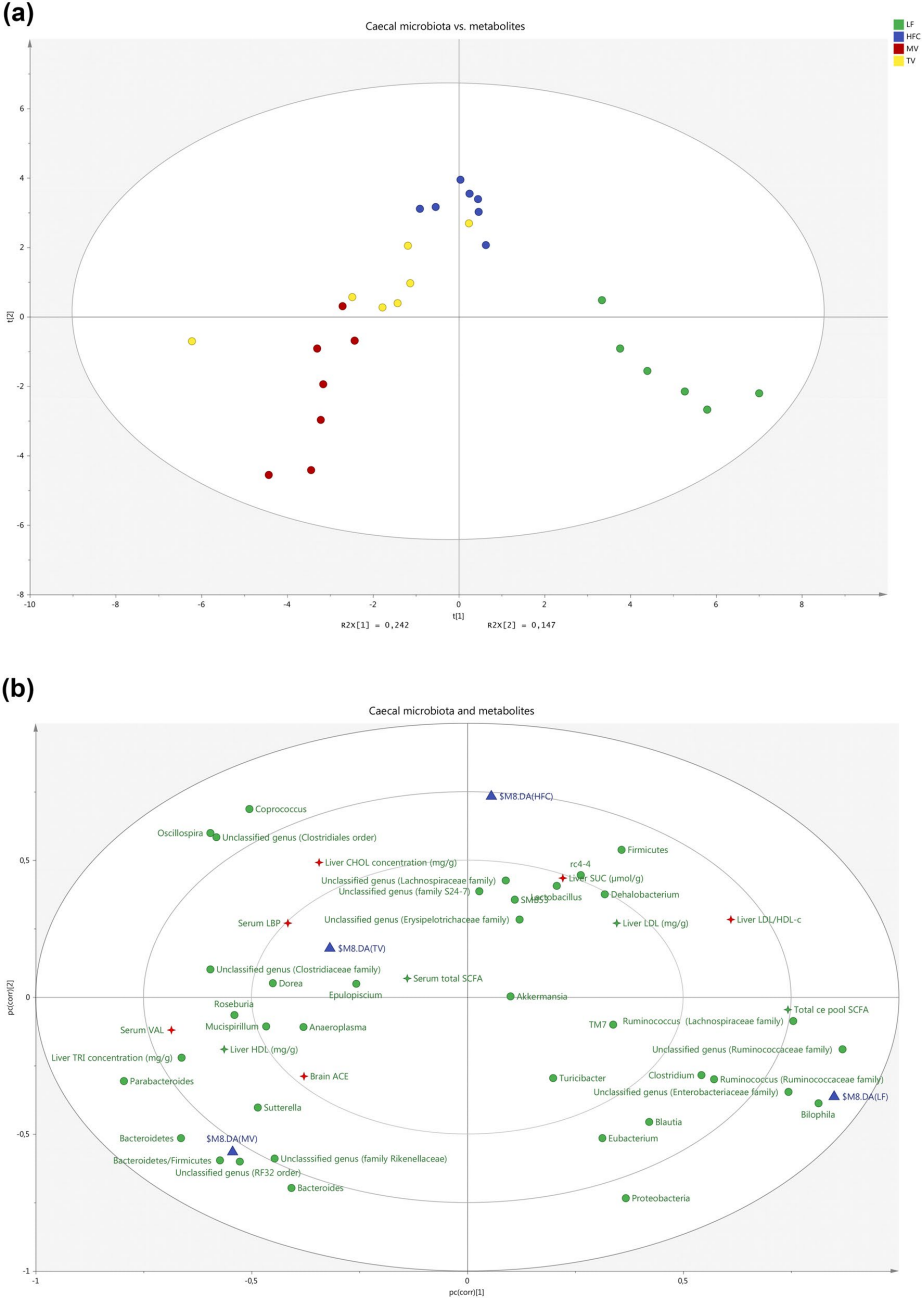
**a** Score scatter plot displays location of groups (marked as triangles) fed a low-fat (LF) diet, a high-fat control (HFC) diet or the HFC diet supplemented with MV or TV for 3 weeks. Each circle represents one rat. **b** Loading scatter plot shows relation between the gut microbiota and parameters in serum (valeric acid and LBP), liver (succinic acid, total cholesterol, LDL-, HDL-cholesterol, and LDL/HDL ratio), and brain (acetic acid). Microbial taxa are shown as green circles, and metabolites as 4-point stars

In Fig. 5, there is an overview of the relation between groups based on microbiota data (at both phylum and genus level) and variables with significance. The different groups are separated in different areas of the loading scatter plot. The HFC group was associated to higher liver succinic acid, total liver cholesterol and LDL/HDL ratio, as well as enriched abundances of Firmicutes and *rc4-4*. Notably, the same values were shown to be lower for the MV group as shown by its opposite direction in the loading scatter plot. In contrast, acetic acid in the brain was higher, as well as serum valeric acid and the abundances of Bacteroidetes and Bacteroidetes/Firmicutes ratio in the group fed MV. The TV group was located between the HFC and MV groups.

## Discussion

### Higher amounts of acetic acid in the brain with monovalerin and trivalerin

Addition of MV and TV to a HFC diet resulted in significantly 1.5-fold increased amounts of acetic acid in the rat brain. Higher levels of acetic acid could also be seen in the serum and liver of rats fed these diets, although not that significant, while the amounts in the caecum of rats were lower. This indicates that caecal acetate can be delivered to the brain, which also has been shown in mice by others [15]. Further, an increased uptake of acetate to the hypothalamus may reduce acute food intake later by up-regulating the expression of appetite suppressing neuropeptides [15]. Our results are in line with this, demonstrating that rats fed MV and TV diets consumed significantly less food compared with the HFC-fed rats during the 3-week study. Notably, in the present study, brain acetic acid was inversely related with liver succinic acid, which has been found to increase in lipopolysaccharide-activated mouse macrophages [21]. Brain acetic acid was also negatively associated with the phylum TM7 and its representative family F16 (bacteria connected to inflammatory diseases [30]) and *rc4-4* (related to high-fat diet-induced obesity [24]), and positively correlated with Tenericutes and *Anaeroplasma*. There are reports that Tenericutes is reduced in aged mice with chronic-low grade inflammation [31], while *Anaeroplasma* is significantly less abundant in obese mice compared with lean mice [32].

The mechanism of the relation between increased amounts of acetic acid in the brain and decreased amounts of succinic acid after consumption of HFC diet supplemented with MV and TV is unknown. However, it is possible that valeric acid acts in a similar way as its analogue, valproic acid, a well-known GABA enhancer, which suppresses succinic acid formation by inhibiting succinic acid synthesizing enzymes [18–20]. Moreover, the decrease in succinic acid may favour the formation of acetyl-coA/acetic acid [33], which may explain the lower formation of succinic acid in this study. The suggested role for valeric acid can also be indicated by following the route of acetic acid (Table 2), which was similar among all groups in serum, but higher in MV and TV groups than HFC group in the liver, to reach significance in the brain (compared with both LF and HFC groups). GABA concentration in human plasma has also been found to increase following oral administration of sodium valproate [34]. There is also evidence that acetic acid, intraperitoneally injected and derived from colonic fermentation, increases hypothalamic GABA level [15]. Therefore, considerable changes of acetic acid in the brain may represent potential intervention to prevent inflammation-related diseases.

### Monovalerin and trivalerin influence levels of succinic acid and valeric acid

The reduction of liver succinic acid by MV and TV is of great interest. Succinic acid, a microbial intermediate in the citric acid cycle, does not accumulate at any substantial levels under normal conditions. However, elevated succinic acid concentrations have been reported in animals subjected to hypertension, metabolic [22] and inflammatory diseases [21]. Due to the enhancement of interleukin-1β production during inflammation, succinate is proposed as an inflammatory signalling molecule, providing similar links between inflammation and cancer [21]. Notably, a high-fat diet increases the proportion of succinic acid at the expense of butyric acid [23, 35]. Consistent with these studies, the ratio of butyric to succinic acid in the liver was higher in MV and TV groups, and very similar to that of the LF group. Furthermore, succinic acid has been reported to increase blood pressure in animals by activating its receptor G-protein coupled receptor 91 (GPR91), proposing a potential role of succinate in hypertension-related diseases, including ischemia and atherosclerosis [36]. From this point, it should be noted that the spleen weight is decreased with MV. In a previous study at our lab, a lower spleen weight was associated with decreased levels of an inflammatory marker MCP-1 in rats fed guar gum [23]. Intriguingly, the spleen plays a pivotal role in modulating hypertension and inflammation via neuro-immune communication [37]. GPR91 is expressed in the spleen, liver, aorta and brain [36, 38]. Expression of a SCFA receptor GPR43 in the spleen was linked to restored defects of microglia in germ-free mice receiving SCFA mixed in drinking water [39]. These data indicate that SCFA can have direct effects on a specific organ and remote impact on other organs via their biochemical crosstalk.

Valeric acid was higher in portal blood of rats in groups fed MV and TV compared with the HFC group, indicating that valeric acid is released from the delivered esters. Interestingly, serum valeric acid was inversely correlated with the LDL/HDL ratio and succinic acid concentration in the liver. Although no significant difference was seen in liver cholesterol between the high-fat groups, rats fed MV and TV diets had lower cholesterol compared with those fed the HFC diet, and very similar to that of the LF group. Not many studies have elucidated the mechanistic action of valeric acid on cholesterol synthesis. However, administration of valeric acid sodium salt has been shown to reduce hepatic cholesterol synthesis in rats, through a proposed mechanism independent of the activity of the enzyme 3-hydroxy-3-methylglutaryl CoA reductase [16]. Thus, a longer experimental time could have provided a more distinct effect on cholesterol induced by MV and TV. In relation to the increase in brain acetic acid by MV and TV, it was shown that valeric acid possesses GABAergic effects by ameliorating neuronal variables in a rat model of dementia [17]. Reductions of GABA currents and GABA_A_ receptors are reported in human Alzheimer diseased brains [40]. Coupled with the effects of succinic and acetic acids, MV and TV can be used as promising supplements to prevent hypertension, high-fat-induced inflammation, or potentially GABA-related neuronal disorders. However, it is important to identify at which concentrations valeric acid is harmless in the blood, since its high concentrations have been found in venous blood of patients with microscopic colitis [41], as well as in faecal samples of celiac disease and obese subjects [42, 43].

### Monovalerin and trivalerin change high-fat-induced microbiota composition

Supplementation of MV and TV to a high-fat diet altered the caecal microbiota composition, with most differences in composition for the MV group. At phylum level, it is interesting that addition of MV into the HFC diet led to an increased ratio of Bacteroidetes-to-Firmicutes. Increases in this ratio have been associated with high-fibre diets in humans and mice [44, 45]. A decrease of this ratio is typically found in genetically *ob*/*ob* obese mice and humans [6] and also in faecal samples of coronary artery disease patients [46]. In addition, the abundance of TM7 was absent in the TV group and low in the MV group. An increased abundance of this phylum is found in inflammatory bowel disease patients [30] and colitis-induced mice [47]. Although accounting for low abundance, Tenericutes distinguished dissimilarity in gut microbiota profile between the LF and the HFC groups. This phylum has been found to increase in rats fed a high-fat diet compared with those on a low-fat control diet in other studies [48].

Further analysis at genus level revealed microbial alteration associated with the changed metabolites in the present study. For instance, the decrease in liver succinic acid was associated with the abundance of the family S24-7. *Bacteroides*, showing higher abundance in MV group, was inversely linked to liver cholesterol. This genus is less abundant and negatively related to waist circumference in obese subjects [43]. The lower liver cholesterol observed in the LF group was also correlated with a decrease of *Oscillospira* and *Dorea*, and an increase of *Bilophila. Oscillospira* has been reported to increase over time in the caecum of rats fed a high-fat diet, whereas a decrease of this genus in the ileum is linked with paracellular permeability [49]. *Dorea* is enriched in high-fat feeding [24, 49], whereas *Bilophila* is increased in low-fat/high sugar diets [50]. Overall, supplementation of MV and TV could be of importance in modulating the high-fat-induced gut microbiota composition towards a less obese and inflammatory state.

### Low-fat group

Some results obtained from the LF group are also worth to be discussed. For instance, although insignificance, LF-fed rats had higher body weight gain compared to rats fed the high-fat diets. This could be due to that the lower energy content of the diet led to an increase in food intake, to satisfy the energy need for physiological requirements. In addition, rapidly digested starch is connected to overconsumption due to lack of energy intake control [51]. Consequently, a low-fat diet with a highly resistant fibre (cellulose) delivers less substrate for the microbiota, inducing unexpected abundances of microbial taxa such as the phylum TM7 in this case. However, it is hardly possible to establish an optimal control diet regarding the microbiota since it is rapidly and dynamically adapted to the diet, and bacterial cross-feeding exists. Therefore, it would perhaps be more relevant if a low-fat diet containing fermentable fibre is used. Apart from these unexpected results, the LF group seemed to be a suitable control for other parameters, especially the lipid profile.

In conclusion, supplementation with MV and TV led to significantly increased brain acetic acid concentrations with simultaneous decrease in liver succinic acid in conventional rats fed a high-fat diet after 3 weeks. These changes were also associated with alterations in the gut microbiota composition. Results from the present study suggest potential use of MV and TV as dietary supplements aimed to counteract or prevent disorders that are accelerated by systemic inflammation, such as obesity and obesity-linked neurodegeneration.

## Electronic supplementary material

Below is the link to the electronic supplementary material.

Supplementary material 1 (DOCX 38 KB)
