# Supplementary material for: Monovalerin and trivalerin increase brain acetic acid, decrease liver succinic acid, and alter gut microbiota in rats fed high-fat diets
Source: Eur J Nutr. 2018 Apr 12;58(4):1545–60. doi: 10.1007/s00394-018-1688-z (PMC6561987; doi:10.1007/s00394-018-1688-z)
Supplement: Supplementary file 1 — Supplementary material 1 (DOCX 38 KB) [file 394_2018_1688_MOESM1_ESM.docx]

# Article title: **Monovalerin and trivalerin increase brain acetic acid, decrease liver succinic acid, and alter gut microbiota in rats fed high-fat diets**

Journal name: European Journal of Nutrition

Author names: Thao Duy Nguyen, Olena Prykhodko, Frida Fåk Hållenius and Margareta Nyman

Food for Health Science Centre

Present affiliation: Lund University, Department of Food Technology, Engineering and Nutrition

Address: Lund University, PO Box 124, SE-221 00, Lund, Sweden

Corresponding author: Thao Duy Nguyen, email: [thao_duy.nguyen@food.lth.se](mailto:thao_duy.nguyen@food.lth.se)

**Supplementary Table S1** Composition of the test diets (g/kg dry weight)

| Ingredients/Diets | **LF** | **HFC** | **MV/TV** |
| --- | --- | --- | --- |
| Casein† | 150.0 | 150.0 | 150.0 |
| DL- Methionine† | 1.2 | 1.2 | 1.2 |
| Butterǂ | 0.0 | 180.0 | 180.0 |
| Rapeseed oil | 50.0 | 50.0 | 50.0 |
| Sucrose | 100.0 | 100.0 | 100.0 |
| Cellulose§ | 50.0 | 50.0 | 50.0 |
| Mineral mixture‖ | 48.0 | 48.0 | 48.0 |
| Vitamin mixture‖ | 8.0 | 8.0 | 8.0 |
| Choline chloride† | 2.0 | 2.0 | 2.0 |
| Ester product | 0.0 | 0.0 | 5.0 |
| Wheat starch¶ | 590.8 | 410.8 | 405.8 |

LF, low-fat, HFC, high-fat control; MV, monovalerin; TV, trivalerin.

† Sigma-Aldrich, St. Louis, MO, USA.

ǂ Arla Foods, Stockholm, Sweden.

§ FMC BioPolymer, Cork, Ireland.

‖ Containing (g/kg): 0.37 CuSO_4_.5H_2_O, 1.4 ZnSO_4_.7H_2_O, 332.1 KH_2_PO4, 171.8 NaH_2_PO4.2H_2_O, 324.4 CaCO_3_, 0.068 KI, 57.2 MgSO_4_, 7.7 FeSO_4_.7H_2_O, 3.4 MnSO_4_.H_2_O, 0.02 CoCl.6H_2_O, 101.7 NaCl, 0.019 chromium (III) chloride and 0.011 sodium selenite (Lantmännen, Stockholm, Sweden).

‖ Containing (g/kg): 0.62 menadione, 2.5 thiamin hydrochloride, 2.5 riboflavin, 1.25 pyridoxine hydrochloride, 6.25 calcium pantothenate, 6.25 nicotinic acid, 0.25 folic acid, 12.5 inositol, 1.25 *p*-aminobenzoic acid, 0.05 biotin, 0.00375 cyanocobalamin, 0.187 retinol palmitate, 0.00613 calciferol, 25 D-*α*-tocopheryl acetate, 941.25 maize starch (Lantmännen, Stockholm, Sweden).

¶ Cargill, Sas van Gent, The Netherlands; the amount varied depending on the ester and fat content of the test diets.

**Supplementary Table S2** Relative gene expression of Cyp7a1, Cyp8b1, and Nr0b2 in the liver of rats fed a low-fat (LF) diet, a high-fat control (HFC) diet or the HFC diet supplemented with MV or TV for 3 weeks

| Genes/Groups | **LF** | | **HFC** | | **MV** | | **TV** | |
| --- | --- | --- | --- | --- | --- | --- | --- | --- |
|  | Mean | SEM | Mean | SEM | Mean | SEM | Mean | SEM |
| *Cyp7a1* | 3.09 | 0.07 | 2.91 | 0.07 | 2.87 | 0.08 | 2.91 | 0.03 |
| *Cyp8b1* | 2.54 | 0.04 | 2.52 | 0.04 | 2.52 | 0.03 | 2.49 | 0.02 |
| *Nr0b2* | 2.90 | 0.04 | 2.83 | 0.04 | 2.86 | 0.05 | 2.88 | 0.03 |

Values are mean ± SEM, n=7

**Supplementary Table S3** Composition of ester products (%, w/w)

|  | **Mono-valerate** | **Di-valerate** | **Tri-valerate** | **Glycerol** | **Etyl-valerate** | **Valeric acid** | **Water** |
| --- | --- | --- | --- | --- | --- | --- | --- |
| **MV** | 46 | 22.4 | 1.6 | 23.7 | 1.6 | 3.3 |  |
| **TV** |  | 2 | 95 |  |  |  | 0.04 |
